# Spike-Timing-Based Computation in Sound Localization

**DOI:** 10.1371/journal.pcbi.1000993

**Published:** 2010-11-11

**Authors:** Dan F. M. Goodman, Romain Brette

**Affiliations:** 1 Laboratoire Psychologie de la Perception, CNRS and Université Paris Descartes, Paris, France; 2 Département d'Etudes Cognitives, Ecole Normale Supérieure, Paris, France; University College London, United Kingdom

## Abstract

Spike timing is precise in the auditory system and it has been argued that it conveys information about auditory stimuli, in particular about the location of a sound source. However, beyond simple time differences, the way in which neurons might extract this information is unclear and the potential computational advantages are unknown. The computational difficulty of this task for an animal is to locate the source of an unexpected sound from two monaural signals that are highly dependent on the unknown source signal. In neuron models consisting of spectro-temporal filtering and spiking nonlinearity, we found that the binaural structure induced by spatialized sounds is mapped to synchrony patterns that depend on source location rather than on source signal. Location-specific synchrony patterns would then result in the activation of location-specific assemblies of postsynaptic neurons. We designed a spiking neuron model which exploited this principle to locate a variety of sound sources in a virtual acoustic environment using measured human head-related transfer functions. The model was able to accurately estimate the location of previously unknown sounds in both azimuth and elevation (including front/back discrimination) in a known acoustic environment. We found that multiple representations of different acoustic environments could coexist as sets of overlapping neural assemblies which could be associated with spatial locations by Hebbian learning. The model demonstrates the computational relevance of relative spike timing to extract spatial information about sources independently of the source signal.

## Author Summary

There is growing evidence that the temporal coordination of spikes is important for neural computation, especially in auditory perception. Yet it is unclear what computational advantage it might provide, if any. We investigated this issue in the context of a difficult auditory task which must be performed quickly by an animal to escape a predator: locating the source of a sound independently of the source signal. Using models, we found that when neurons encode auditory stimuli in spike trains, the location-specific structure of binaural signals is transformed into location-specific synchrony patterns. These patterns are then mapped to the activation of specific neural assemblies. We designed a simple neural network model based on this principle which was able to estimate both the azimuth and elevation of unknown sounds in a realistic virtual acoustic environment. The relationship between binaural cues and source location could be learned through a supervised Hebbian procedure. The model demonstrates the computational relevance of relative spike timing in a difficult task where spatial information must be extracted independent of other dimensions of the stimuli.

## Introduction

Animals must be able to rapidly estimate the location of the source of an unexpected sound, for example to escape a predator. This is a challenging task because the acoustic signals at the two ears vary with both the source signal and the acoustic environment, and information about source location must be extracted independently of other causes of variability. Psychophysical studies have shown that source localization relies on a variety of acoustic cues such as interaural time and level differences (ITDs and ILDs) and spectral cues [1]. At a neuronal level, spike timing has been shown to convey information about auditory stimuli [2], [3], and in particular about source location [4], [5]. Although it is well accepted that ITDs can be extracted from phase-locked responses, it is unknown how information beyond this could be extracted from the spike timing of neurons. In addition, the potential computational advantages of a spike timing code in this task are unclear.

The sound S produced by a source propagates to the ears and is transformed by the presence of the head, body and pinnae, and possibly other aspects of the acoustic environment (such as reflections). It results in two linearly filtered signals F_L_*S and F_R_*S (linear convolution) at the two ears, where the filtering depends on the relative position of the head and source. Because the two signals are obtained from the same source signal, the binaural stimulus has a particular structure, which is indicative of source location. When these signals are transformed into spike trains, we expect that this structure is transformed into synchrony patterns. Therefore, we examined the synchrony patterns induced by spatialized sounds in neuron models consisting of spectro-temporal filtering and a spiking nonlinearity, where binaural signals were obtained using a variety of sound sources filtered through measured human head-related transfer functions (HRTFs). We then complemented the model with postsynaptic neurons responding to both sides, so that synchrony patterns induced by binaural structure resulted in the activation of location-specific assemblies of neurons. The model was able to precisely encode the source location in the activation of a neural assembly, in a way that was independent of the source signal.

Several influential models have addressed the mechanisms of sound localization at an abstract level [6]–[9]. Considerable progress has also been realized in understanding the physiological mechanisms of cue extraction, in particular neural mechanisms underlying ITD sensitivity [10]–[14]. These studies mostly used simplified binaural stimuli such as tones or noise bursts with artificially induced ITDs. Several purely computational models [15]–[17] address the full problem of sound localization in a virtual acoustic environment with realistic sounds, although these do not suggest how neurons might perform this task. Here we propose a binaural neural model that performs the full localization task in a more realistic situation, based on the idea that synchrony reflects structural properties of stimuli, which in this setting are indicative of source location.

## Results

### Synchrony patterns induced by location-dependent filtering

**Figure 1:**
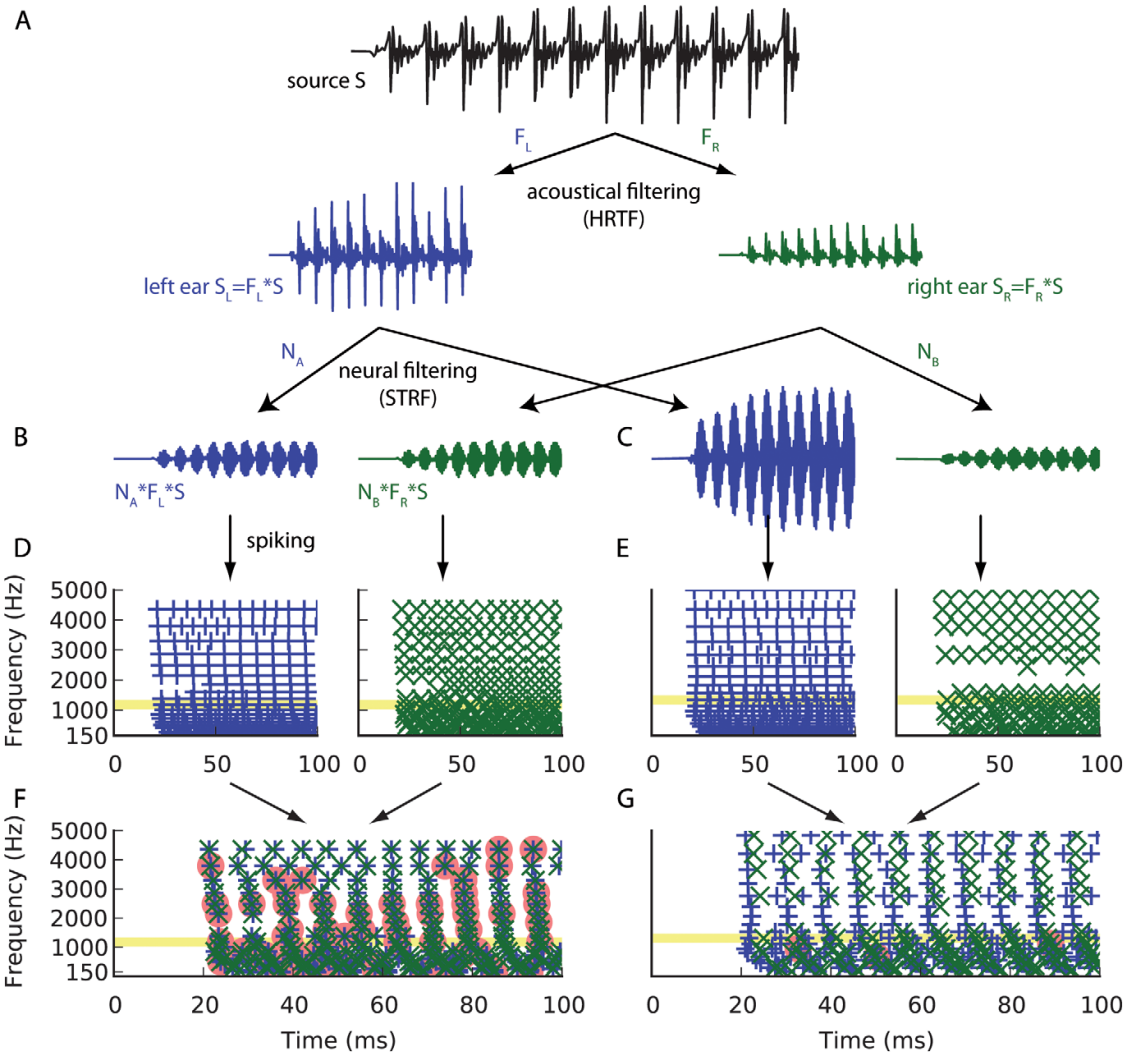
Synchrony patterns induced by location-dependent filtering. The left hand panels (A, B, D, F) show the signal pathway for a set of neurons tuned to the presented location of the sound, and the right hand panels (A, C, E, G) show the pathway for neurons not tuned to the presented location. (A) The sound source S propagates to the left ear (blue) and right ear (green) and is acoustically filtered by location-dependent HRTFs. (B, C) Signals resulting from filtering through the spectro-temporal receptive fields of two pairs of monaural neurons tuned at 1 kHz. The source location is in the synchrony receptive field of the first neuron pair (B). (D, E) Spike trains produced after neural filtering, for neurons tuned at frequencies between 150 Hz and 5 kHz. The signals shown in B and C correspond to the spike trains highlighted in yellow. The source location is in the synchrony receptive field of each frequency-specific neuron pair in panel D but not in panel E. (F, G) Spike trains from the left and right channels are reproduced here superimposed. Postsynaptic neurons that receive coincident inputs from their two presynaptic neurons produce spikes (red patches). The neural assembly in F is tuned to the presented location whereas the assembly in G is not.

Consider a sound source located at azimuth θ and elevation ϕ. The signal S(t) arrives at the two ears as two linearly filtered signals S_L_ = HRTF_L_(θ,ϕ)*S and S_R_ = HRTF_R_(θ,ϕ)*S (Figure 1, A). Other aspects of the acoustic environment such as reflections and distance would also impact the binaural signal, but their effect can always be expressed with linear filters. In general, the signals at the two ears are filtered versions of the source signal, where the filters are determined by the relative positions of the head and source in the acoustic environment. What are the correlates of this filtering at neural level? Let us consider a neuron A which responds to sounds from the left ear. A simplified way to model its response is to consider that the sound is transformed into spike trains after filtering through the neuron's spectro-temporal receptive field N_A_ (Figure 1, B–C), that is, the filtered signal N_A_*S_L_ = N_A_* HRTF_L_(θ,ϕ)*S is followed by a spiking nonlinearity (Figure 1, D–E). Since we are interested in precise spike timing, we consider that the spiking nonlinearity is represented by a neuron model, e.g. integrate-and-fire or more complex models, rather than by a Poisson process. The response of a neuron B to sounds from the right ear would similarly be modeled as spike trains produced from the signal N_B_*S_R_ = N_B_* HRTF_R_(θ,ϕ)*S.

None of these individual neurons expresses spatial tuning, but we now consider the pair of neurons A and B and ask ourselves when these two neurons fire in synchrony. More precisely, we define the *synchrony receptive field* of this neuron pair as the set of stimuli that induce synchronous firing in these neurons. Synchrony occurs when the input signals to the two neurons match, that is, when the following identity is met: N_A_* HRTF_L_(θ,ϕ) = N_B_* HRTF_R_(θ,ϕ) (Figure 1, left column). In the left column of Figure 1, the synchrony receptive fields of pairs of monaural neurons contain the presented location, while in the right column they do not. This identity expresses the condition that the combinations of acoustical and neural filtering match on both sides. Thus, the synchrony field is defined independently of the source signal S, as long as the signal contains energy in the neurons' receptive fields. It is a set of pairs of acoustical filters (HRTF_L_, HRTF_R_), which defines a spatial field: synchrony between the two neurons signals spatial information independently of the source signal. For example, if neuron A has receptive field N_A_ = HRTF_R_(θ*,ϕ*) and neuron B has receptive field N_B_ = HRTF_L_(θ*,ϕ*), then the synchrony field of the pair contains the location (θ*,ϕ*). This example corresponds to a recent signal processing method designed by MacDonald [16], which was found to be very accurate in estimating the azimuth of a sound source. The same holds true if the receptive fields are band-pass filtered in the same frequency band, i.e., N_A_ = K*HRTF_R_(θ*,ϕ*) and N_B_ = K*HRTF_L_(θ*,ϕ*) (Figure 1, D–E). Thus, any given location will elicit a specific pattern of synchrony in a way that is independent of the signal S. Now consider a postsynaptic neuron that receives inputs from these two neurons A and B: if it is sensitive to the relative timing of its inputs then it will fire preferentially when the two inputs are synchronous, that is when the stimulus is in the synchrony receptive field of its inputs (Figure 1, F–G).

**Figure 2:**
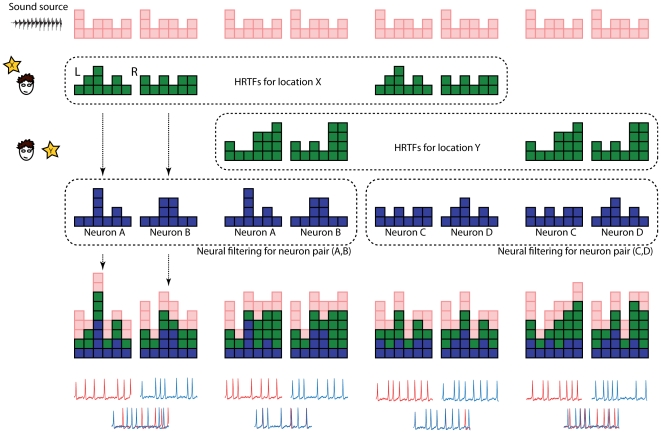
Relationship between synchrony receptive field and source location. We represent each signal or filter by a set of columns, which can be interpreted as the level or phase of different frequency components (as in a graphic equalizer). In the first two columns, the sound source (pink) is acoustically filtered through the pairs of HRTFs corresponding to location X (green), then filtered through the receptive fields of neurons A and B (blue), and resulting signals are transformed into spike trains (red and blue traces). In this case, the two resulting signals are different and the spike trains are not synchronous. In the next two columns, the source is presented at location Y, corresponding to a different pair of HRTFs. Here the resulting signals match, so that the neurons fire in synchrony: location Y is in the synchrony receptive field of neuron pair (A,B). The next 4 columns show the same processing for locations X and Y but with a different pair of neurons (C,D). In this case, location X is in the synchrony receptive field of (C,D) but not location Y. When neural filters are themselves HRTFs, this matching corresponds to the maximum correlation in the localization algorithm described by MacDonald [16]. When neural filters are only phase and gain differences in a single frequency channel, the matching would correspond to the Equalisation-Cancellation model [18]–[20].

To better understand how the neural pattern of synchrony encodes source location, consider that each signal or filter in the processing chain is described by a set of columns of various heights, in the same way as a graphic equaliser (Figure 2): each column represents the level or phase of an individual frequency component. In Figure 2, signals are in pink, acoustical filters (HRTFs) in green and neural filters (receptive fields) in blue. Combining two filters (or filtering a signal) corresponds to adding each column of the first filter on top on the corresponding column of the second filter. The first two columns illustrate the case when the source location X is not in the synchrony receptive field of the neuron pair (A, B): when the signal is combined with the left HRTF and with the receptive field of neuron A, it does not match the combined signal on the other side (combination of signal, right HRTF and receptive field of neuron B). Thus neurons A and B do not fire in synchrony. On the other hand (next two columns), when location Y is presented, the two signals match and the neurons fire in synchrony, which can make a postsynaptic binaural neuron fire. From this illustration, it clearly appears that the two signals would also match if the signal S (pink cubes) were different, and that the synchrony receptive field contains more than a single pair of HRTFs – therefore higher spatial selectivity requires several different neuron pairs. The next two columns illustrate the synchrony receptive field of two other neurons C and D, which contains location X but not Y. Thus location X induces synchrony between A and B, while location Y induces synchrony between C and D. Therefore the pattern of synchrony indicates the location of the sound source, independently of the source signal S. This idea of binaural matching was recently implemented in a signal processing method to estimate the azimuth of a sound source [16], with excellent performance. In that algorithm, the set of “neural filters” corresponds to all possible HRTFs (no band-pass filtering) and the “synchrony pattern” is reduced to a single neuron pair, where synchrony and coincidence detection are replaced by maximum correlation. On the other hand, if neural filters consist only of gains and delays, then the binaural matching would correspond to the Equalisation-Cancellation model [18]–[20], where synchrony and coincidence detection are replaced by cancellation.

**Figure 3:**
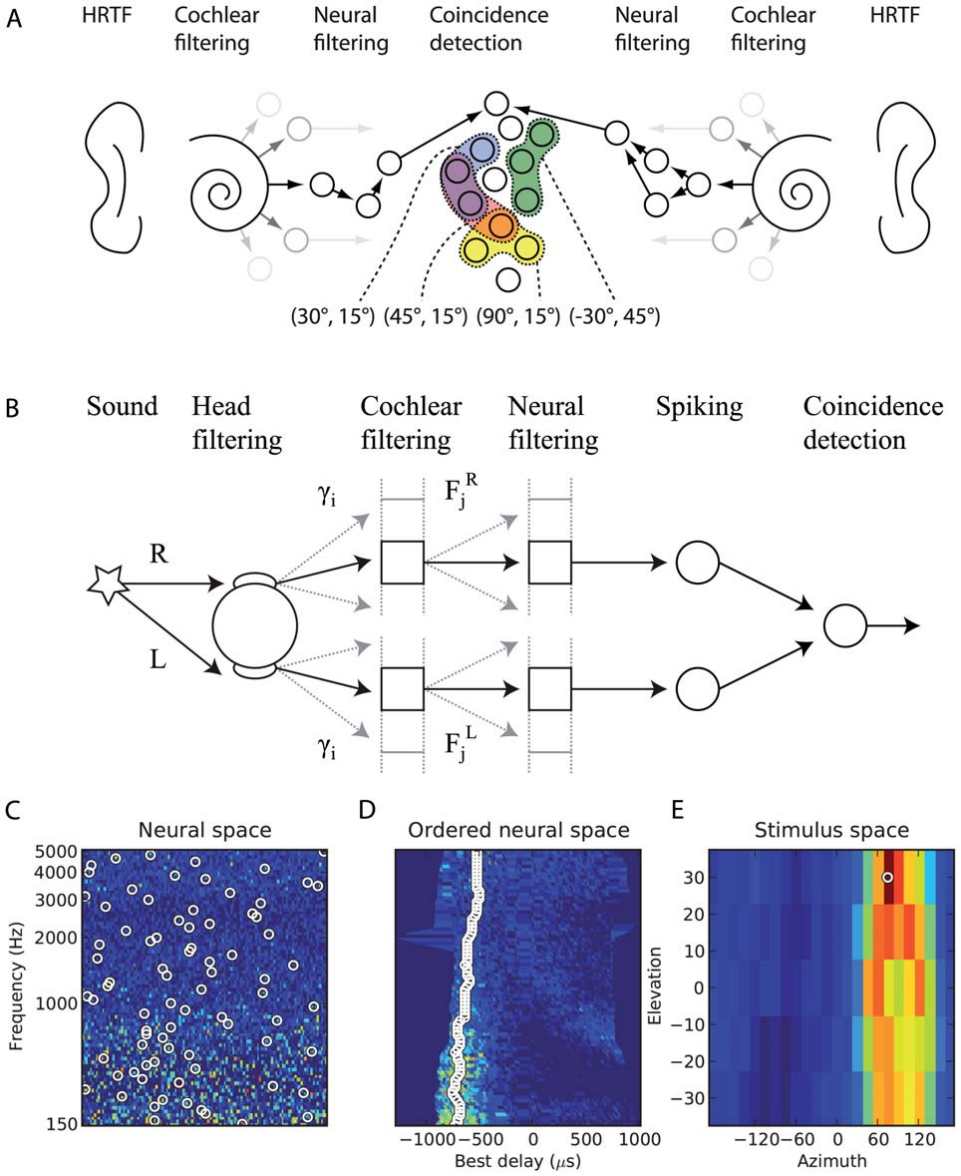
Overview of the model. (A) The source signal arrives at the two ears after acoustical filtering by HRTFs. The two monaural signals are transformed along the auditory pathway (decomposition into multiple frequency bands by the cochlea and further neural transformations) and transformed into spike trains by monaural neurons. These spike trains converge on neurons which fire preferentially when their inputs are coincident. Location-specific synchrony patterns are thus mapped to the activation of neural assemblies (shown here as (azimuth, elevation) pairs). (B) Detailed model architecture. Acoustical filtering (R,L) is simulated using measured HRTFs. The resulting signals are filtered by a set of gammatone filters γ_i_ with central frequencies between 150 Hz and 5 kHz, followed by additional transformations (“neural filtering” F_j_^L/R^). Spiking neuron models transform these filtered signals into spike trains, which converge from each side on a coincidence detector neuron (same neuron model). The neural assembly corresponding to a particular location is the set of coincidence detector neurons for which the synchrony field of their inputs contains that location (one pair for each frequency channel). (C) Model response to a sound played at a particular location. Colors represent the firing rate of postsynaptic neurons, vertically ordered by preferred frequency (the horizontal axis represents a dimension orthonogal to the tonotopical axis). The neural assembly that encodes the presented location is represented by white circles. (D) Same as in (C), but neurons are ordered by preferred interaural delay. (E) Total response of all neural assemblies to the same sound presentation, as a function of their assigned location. The most activated assembly encodes for the presented source location.

**Figure 4:**
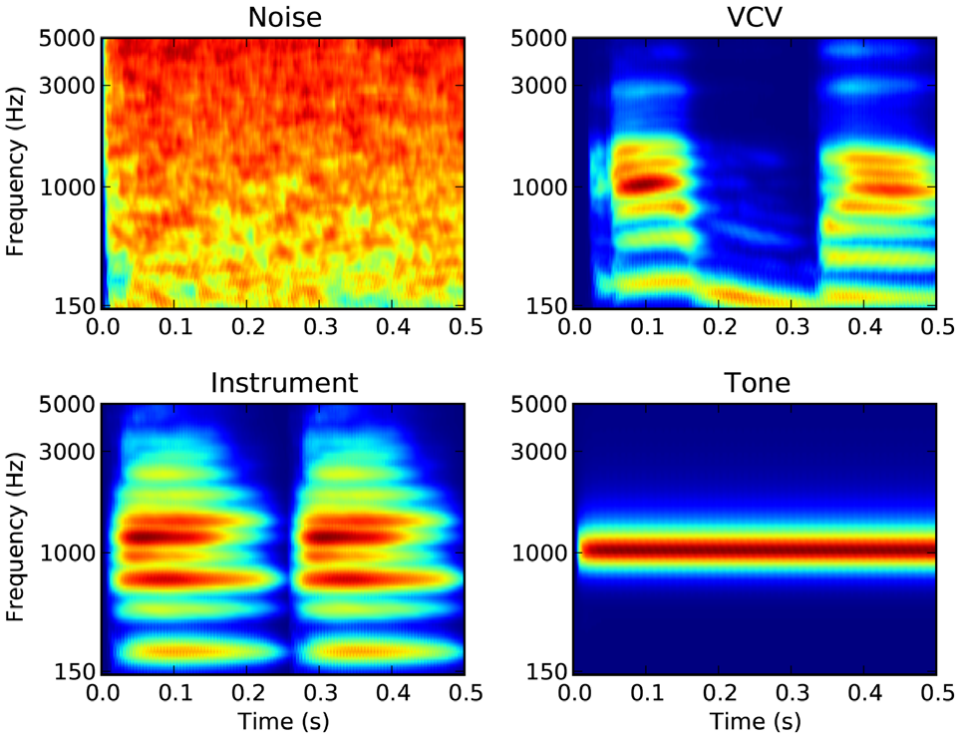
Cochleograms of test sounds used in simulations: white noise; vowel-consonant-vowel (VCV); instruments; pure tones (between 150 Hz and 5 kHz, uniformly distributed in ERB scale). The cochleograms show the output of the gammatone filters used in the model, half-wave rectified and low-pass filtered [56].

**Figure 5:**
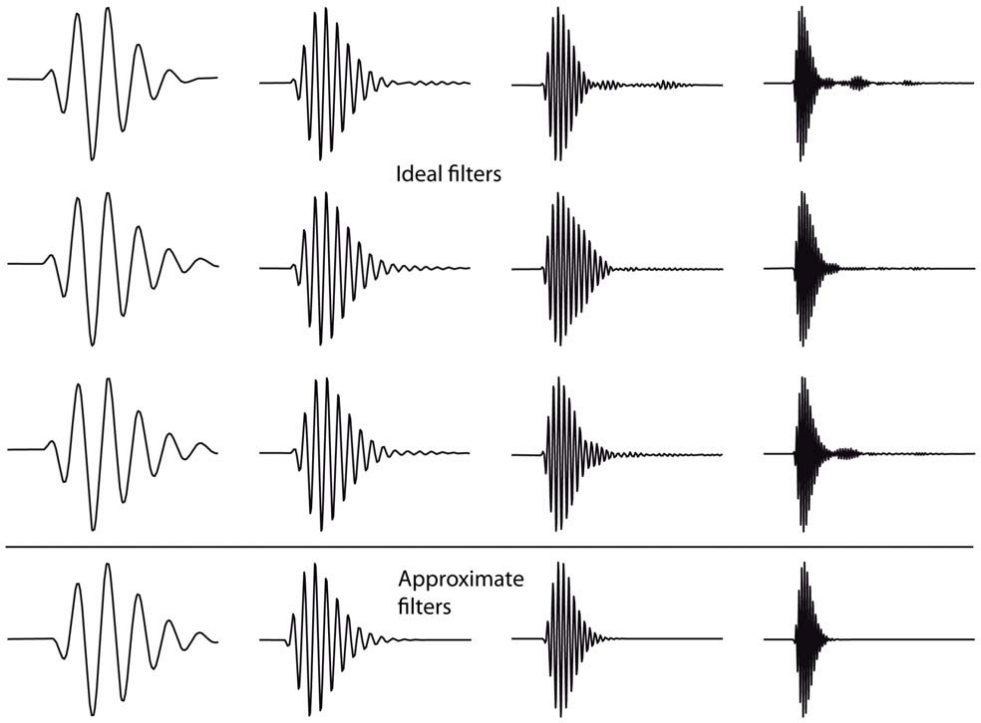
Examples of neural filters in the ideal and approximate model, which are band-pass filtered HRTFs. Each filter is 45 ms long. The central frequency varies between columns, while the HRTFs vary between rows in the first three rows. The resulting filters are similar to gammatone filters (shown in the last row), but not identical (see for example the differences in envelope within the first column, and within the last column).

A given source location, then, will activate a specific assembly of postsynaptic neurons – all those neurons for which the synchrony field of their inputs contains that location – so that source locations are mapped to the activation of (possibly overlapping) neural assemblies (Figure 3A). To test this principle, we simulated a virtual acoustic environment using measured HRTFs and implemented a spiking network model which responded to the binaural signals as described above (Figure 3). A variety of short sounds (noise bursts, musical instruments, voices, tones; Figure 4) was filtered through pairs of human HRTFs to reproduce the natural acoustical filtering of sounds due to the presence of the head, body and pinnae. Neural filtering was modeled as band-pass filtering followed by some additional linear transformations (Figure 3B). These were either all the transformations that we may possibly need to represent all locations, i.e., the complete set of HRTFs (the *ideal model*), or only delays and gains (the *approximate model*). The ideal model should not be taken to imply that the auditory system actually implements HRTF filtering, which would be physiologically unreasonable, but that the neural receptive fields may correspond to band-pass filtered HRTFs. Figure 5 shows 12 examples of such neural filters, which look very similar to gammatone filters, except for slight changes in their envelopes. Nevertheless, these filters may be too diverse to be represented in the auditory system, which motivated the approximate model: in narrow frequency bands, filters can be well approximated by a more restricted set including a range of gains and delays. The resulting signals were then transformed into spike trains with noisy spiking models (mostly integrate-and-fire neurons, with more complex models in one case). Finally, postsynaptic neurons received inputs from two monaural neurons, and were modeled in the same way (Figure 3B). For each source location, we assigned a neural assembly by selecting all the neurons for which the synchrony field of the inputs contained that location, making one neuron per frequency channel (Figure 3C,D). The output of the model was the assigned location of the maximally activated assembly (Figure 3E), and the goal was to predict the actual location of the source. The ideal model is conceptually close to a recent signal processing method designed by MacDonald [16], while the approximate model resembles the Equalisation-Cancellation model [18]–[20], although these two techniques are not neuron models (and the former was only applied on the broadband signal rather than in multiple frequency bands). We describe their relationship with our model in more detail in the Discussion.

### Estimation results for the ideal model

**Figure 6:**
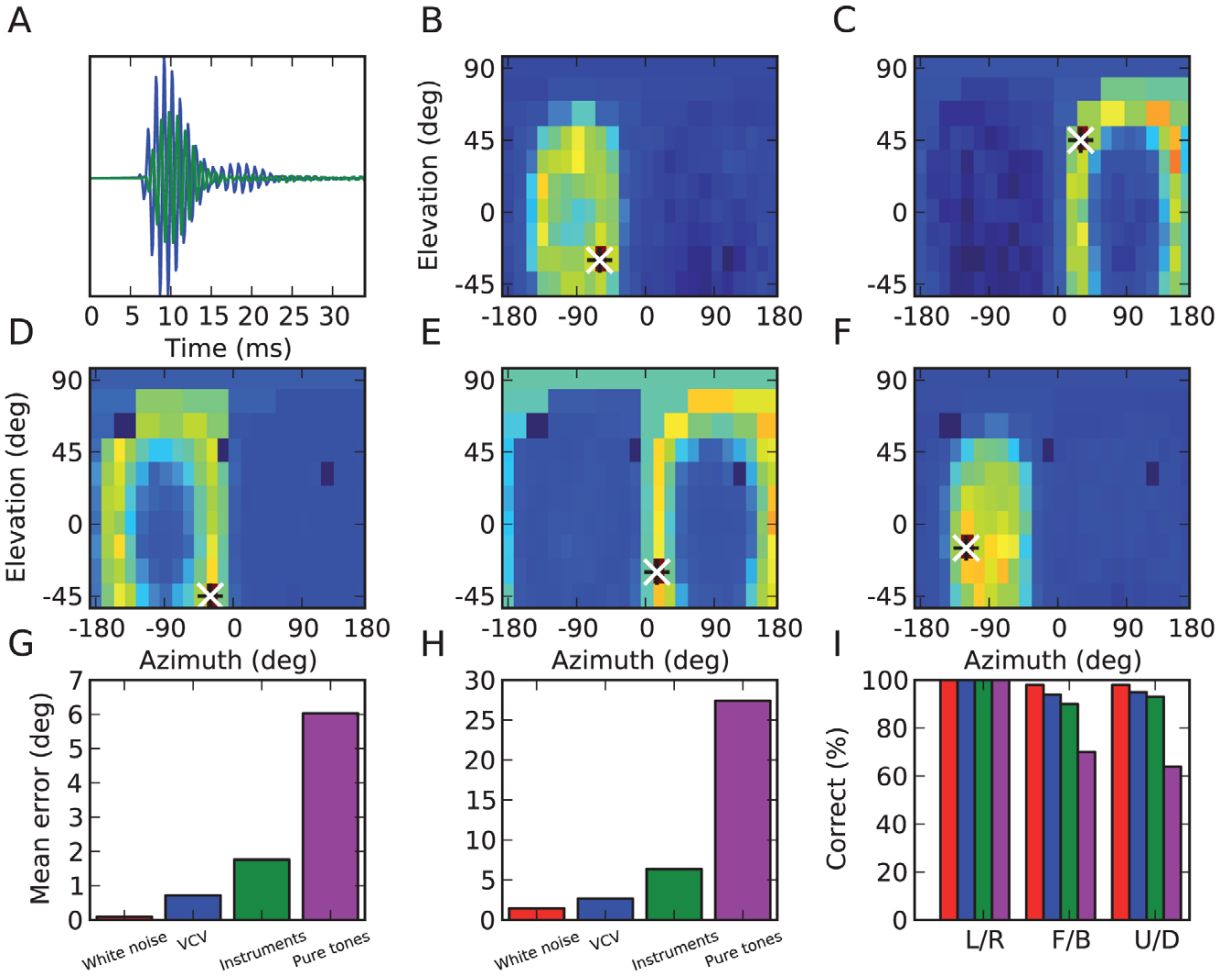
Estimation results in the ideal model. (A) Left (blue) and right (green) head-related impulse responses (HRIR) for a particular location passed through a gammatone filter. (B, C) Activation of all location-specific neural assemblies for two particular source locations, represented as a function of their assigned location. The black+shows the sound location and the white x shows the model estimate (maximally activated assembly). (D–F) Spatial receptive fields of three neural assemblies, i.e., total activation as a function of source location. (G) Mean error in azimuth estimates for white noise (red), vowel-consonant-vowel (blue), musical instruments (green) and pure tones (magenta). Front/back confusions do not contribute to azimuth errors in this panel. (H) Mean error in elevation estimates. (I) Categorization performance discriminating left and right (L/R), front and back (F/B) and up and down (U/D).

**Figure 7:**
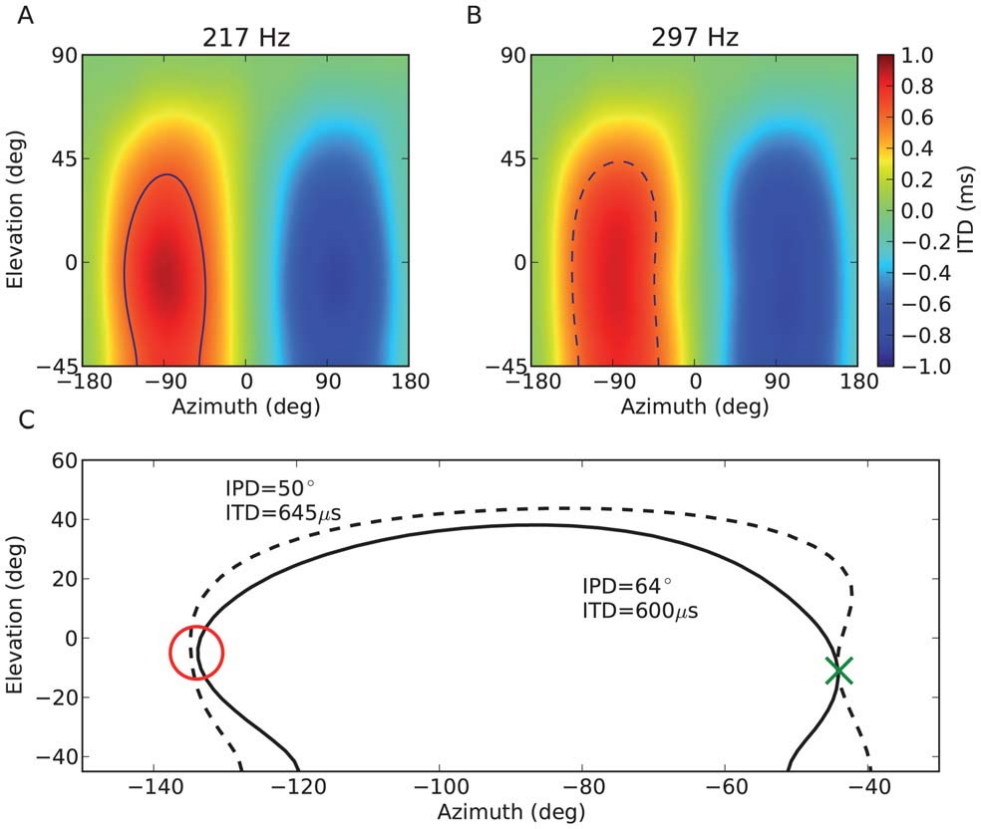
Illlustration of how the model can estimate both azimuth and elevation. (A) ITD measured at 217 Hz as a function of source location. When the sound is presented at azimuth −45° and elevation −10°, the ITD is consistent with all locations shown by the solid curve. For a spherical head, this curve corresponds to the “cone of confusion”. (B) ITD measured at 297 Hz vs. source location. The pattern is similar but quantitatively different from ITDs measured at 217 Hz (A), because sound diffraction makes ITDs frequency-dependent [21]. The ITD at location (−45°,−10°) is consistent with all locations shown by the dashed curve. (C) When the sound includes frequency components at 217 Hz and 297 Hz and ITDs can be measured in both channels, source location is unambiguously signaled by the intersection of the two level lines (green cross), corresponding to the ITD measured at the two frequencies. The red circle shows that this intersection resolves a potential front-back confusion.

Figure 6B and C show the activation (total spike count) of all location-specific neural assemblies in the ideal model for two particular sound presentations, at locations indicated by black crosses. In both examples, the assigned location of the maximally activated assembly (indicated by white crosses) is indeed the actual source location. Although in these figures we represented model outputs on a map, this topographical representation is not present in the model itself. Figure 6D–F shows the activation of three particular neural assemblies as a function of source location. It appears that these assemblies are spatially tuned in that they fire more when the source is at their assigned location. The spatial receptive fields of these assemblies also reveal ring-like structures: these correspond to the cones of confusion [1], where the distances to the two ears are constant, so that interaural cues are very similar (the rings correspond to circles around the approximate symmetry axis that goes through the two ears). If the head were perfectly spherical, there would be no way to distinguish between these locations, but the model does so thanks to the irregular shape of the head and to the presence of the body (see also Figure 7).

Quantitatively, for the ideal model with 80 frequency channels, the average estimation error for white noise was almost zero degrees for azimuth (Figure 6G) and 1.5 degrees for elevation (Figure 6H). Other types of sounds did not have as much power in all frequency bands (Figure 4), and consequently the estimation error was larger for these sounds, but remained very small for speech (1 degree azimuth and 3 degrees elevation) and musical instruments (2 degrees azimuth and 6 degrees elevation), even though they were not used to build the model. This performance for previously unknown sounds is the key property we were expecting to see in the model, as the synchrony patterns are location-specific and independent of the source signal. The error was substantially higher for pure tones, particularly for elevation (6 degrees azimuth and 28 degrees elevation, which is close to the chance level of 36 degrees given the distribution of source locations). This is not surprising since for high frequencies the ITD cues are ambiguous due to periodicity, and for low frequencies ILD cues are very weak, giving only one dimension in the binaural cues.

Not surprisingly, the model could correctly categorize the sound as coming from the left or right (100% success rate for all types of sounds), but it also performed well in more difficult categorization tasks: discriminating between front and back (70% for pure tones and 90–98% for other sounds), and between up and down (64% for pure tones and 93–98% for other sounds), as shown in Figure 6I. This indicates that different neural assemblies were activated for front and back locations and that the model was thus able to exploit small specific interaural differences, as we show below.

It might be surprising that the model can estimate elevation and even discriminate between front and back while it only uses binaural cues. Figure 7 explains how across-frequency integration (i.e., looking at the spatial tuning of neural assemblies rather than that of individual neurons) allows the model to estimate both azimuth and elevation. The mechanism is illustrated with ITD cues but the same holds for ILD cues. When the ITD is estimated in a fine frequency band, it varies with frequency for the same source location, because of sound diffraction by the head [21]. Specifically, it is larger at lower frequencies. Because the head is not spherical, this frequency-dependence of ITDs is location-specific. When the ITD is observed is a single frequency band, it is consistent with many possible locations (Figure 7A, solid curve), because two dimensions (azimuth, elevation) are mapped to a single one (ITD). In a spherical head model, these possible locations form the cone of confusion [1]. When the ITD is observed in another frequency band, it is also consistent with a whole set of locations (Figure 7B, dashed curve), but this set is frequency-dependent. Therefore if the sound contains the two frequency components, the intersection of the two sets of possible locations is a single point corresponding to the true location of the source (Figure 7C). In other words, if source location is two-dimensional, it can be estimated using two independent observations. Pure tones cannot benefit from this disambiguation, consistently with the poor performance seen in Figure 6G–H (magenta bars). There is experimental evidence that humans can indeed use binaural cues to estimate elevation [22], which we comment on in the Discussion.

### Estimation results for the approximate model

**Figure 8:**
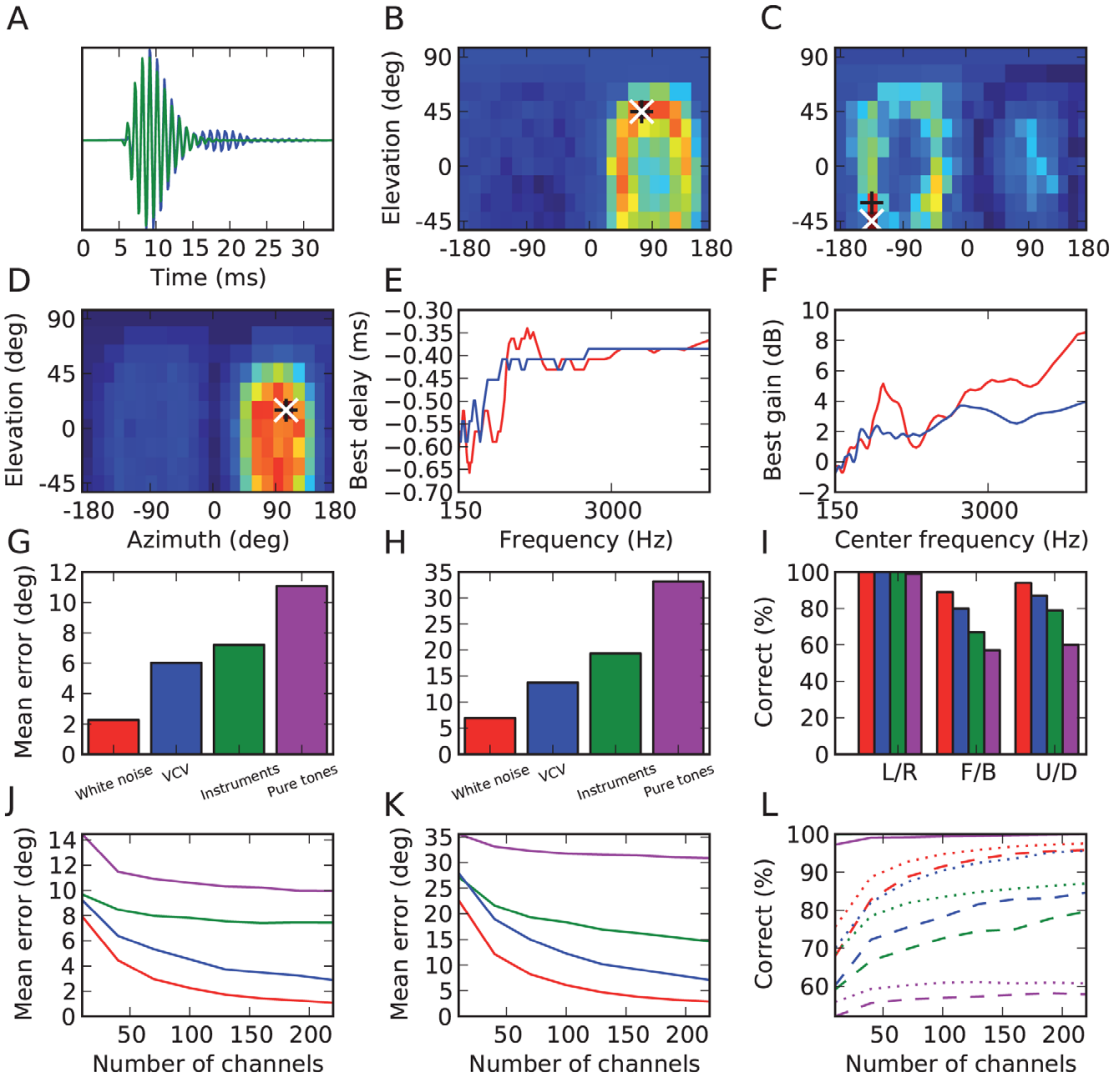
Estimation results in the approximate model. (A) Comparison of a gammatone-filtered HRIR (blue) and an approximate filter (green; gammatone with best delay and gain). (B, C) Activation of neural assemblies for two particular source locations, as in Figure 6. (C) shows a mistake of the model. (D) Spatial receptive field of a particular neural assembly, as in Figure 6. (E) Preferred interaural delay vs. preferred frequency for neurons in two assemblies tuned to locations differing only by a front-back reversion. (F) Interaural gain difference vs. preferred frequency for the same assemblies. (G–I) Performance of the model, as in Figure 6. (J–L) Estimation results as a function of the number of frequency channels used. Simulations were all performed using 240 channels. To obtain estimates of the error using a smaller number of channels while keeping the same frequency range, a randomly chosen subset of the 240 channels was chosen. Error estimates are averaged over many such random choices. (J) Mean error in azimuth estimates for white noise (red), vowel-consonant-vowel sounds (blue), instruments (green) and pure tones (magenta). (K) Mean error in elevation estimates. (L) Categorization performance discriminating left and right (solid), front and back (dashed) and up and down (dotted). For all classes of sounds except the pure tones, the left/right categorization performance is 100% for all points.

It might be unrealistic to assume that the receptive fields of auditory neurons have so much diversity as to include all possible HRTFs. Besides, it is not straightforward to see how the auditory system could learn these acoustical filters. To address this issue, we tested an *approximate model* in which neural filtering consisted only of band-pass filtering with various gains and delays, which could be produced by many mechanisms: axonal or dendritic propagation, inhibition, voltage-gated conductances, etc. Within fine frequency bands, such simple transformations can approximate but not completely match HRTF filtering (Figure 8A) and therefore performance might be expected to drop. However, we found that the location-dependent responses of neural assemblies were very similar to those seen in the ideal model, but slightly less specific (Figure 8B–D). Quantitatively, estimation errors were still small, although not as much as in the ideal model (2 to 7 degrees azimuth and 7 to 20 degrees elevation, excluding pure tones; Figure 8 G–I and Supplementary Figure S1). In this approximate model, it might be thought that binaural neurons perform a cross-correlation of delayed monaural inputs, as in the classical Jeffress model [6]. However, because the inputs to these neurons are precise spike trains rather than Poisson processes, the operation that they perform is more accurately described as a similarity operation (firing when the inputs are similar) than as a cross-correlation. In particular, this operation includes level differences as well as timing differences. We comment on this issue in the Discussion.

In the approximate model, neurons in a location-specific assembly can be fully described by their preferred frequency, interaural delay and gain difference (in log scale). Figure 8E shows the preferred delay of neurons as a function of their characteristic frequency for two neural assemblies tuned to the same location but with front and back reversed, and Figure 8F shows the preferred interaural level difference as a function of frequency. It appears that the preferred delay is approximately constant at high frequencies but irregular at low frequencies (<1 kHz). By construction, the preferred delay corresponds to the ITD in the neuron's frequency channel at these locations, as measured as the peak of crosscorrelation of the band-passed filtered HRTFs. These frequency-dependent patterns are consistent with previous measurements in HRTFs [21], [23] and with theoretical predictions: for high frequencies, acoustical waves behave as light rays and the ITD is determined by the difference in the shortest paths from the source to the ears, but for low frequencies (with wavelength larger than the size of the head) sound propagation is governed by diffraction, which predicts larger and frequency-dependent ITDs [21]. The interesting consequence for sound localization is that the frequency-dependent pattern of ITDs is location-specific, and is therefore a cue for both azimuth and elevation, and can also be used to discriminate between front and back, as is illustrated in Figure 7. Psychophysical studies showed that, indeed, head diffraction and torso reflections provide elevation cues even when pinnae cues are absent [22].

The estimation error decreased as the number of frequency channels in the model was increased (Figure 8, J–L). For example, for white noise, the estimation error in azimuth was halved using 240 channels instead of 80 (Figure 6A). Except for pure tones, the performance did not seem to have converged to an asymptotic value, so we expect the error to be even smaller with more channels. A human cochlea has 3,000 inner hair cells, of which around 1800 have characteristic frequencies between 150 Hz and 5 kHz. For pure tones, the performance did appear to be approaching an asymptotic value, which is not surprising as there are limitations in the available acoustical cues.

In many previous studies, possible source locations were constrained in the horizontal plane (for example [16]). For comparison, we show in Supplementary Figure S2 the estimation error of the model in this case (with 80 frequency channels). The performance was significantly better, especially for the ideal model, which made zero errors except for pure tones (which provide ambiguous information at high frequencies).

### Robustness

**Figure 9:**
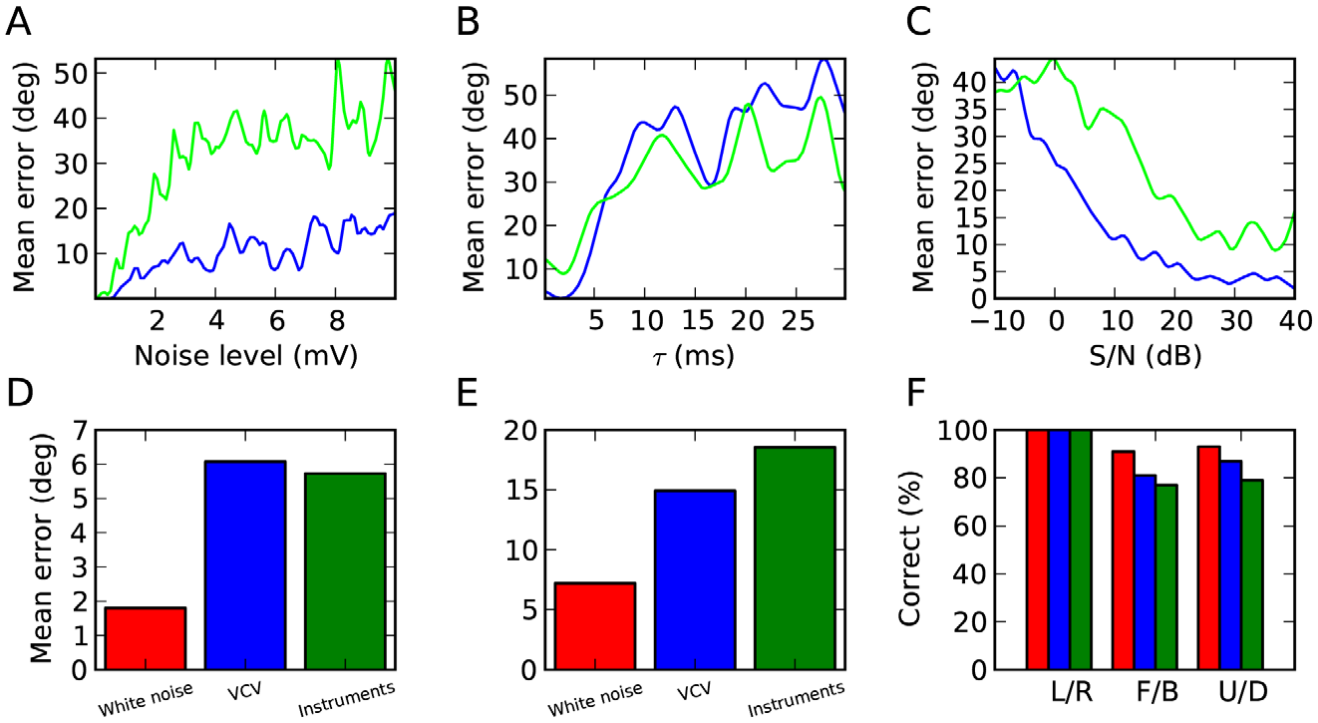
Robustness of the model. (A) Mean error in azimuth (blue) and elevation (green) as a function of the level of intrinsic noise in the model, measured as the standard deviation of the membrane potential. (B) Mean error as a function of the membrane time constant of coincidence detector neurons. (C) Mean error as a function of the signal to noise ratio, with uncorrelated white noise in both ears. (D, E, F) Performance of the model using AdEx neurons in place of LIF neurons (as in Figure 6).

The model relies on selective synchronization and sensitivity to synchrony, which might require specific neural properties, such as low intrinsic noise and short membrane time constant. Figure 9A shows how the estimation error depends on the level of intrinsic neuronal noise in the model. It appears that the performance in azimuth estimation is very robust to noise, and that elevation estimates are reasonably accurate with a noise level up to about 2 mV (standard deviation of the membrane potential). Since the model must be able to resolve submillisecond differences in spike timing, we expected that the membrane time constant of neurons should be small. The results we previously showed were obtained with a membrane time constant of 1 ms, and Figure 9B shows that the model gave reasonable estimates up to about 4 ms, which is not unreasonably short for auditory neurons [24]–[26]. Since the models did not explicitly include voltage-gated conductances [27] and coordinated inhibition [28], which both shorten the integration time constant, this value should be understood as the effective time constant that accounts for these effects. When the time constant was larger than 10 ms, performance was close to chance level, which indeed confirms that the model relied on precise spike timing.

In all previous figures, the performance of the model was only tested in quiet, although physiological noise was included in the neural models. In Figure 9C, we added uncorrelated white noise to the left and right signals and we tested how accurately the approximate model could localise white noise (500 ms). It should be stressed that source locations were not constrained to the horizontal plane, but that the model was nonetheless robust to moderate levels of distracting noise.

All previous results were obtained with simple integrate-and-fire models. However the model is based on the principle that neurons synchronize when their inputs are similar, which should be mostly independent of the specific way in which these inputs are transformed into spike trains. We checked this idea by replacing the neuron models by adaptive exponential integrate-and-fire models tuned to regular spiking cortical cells [29]. This model can predict the spike trains of cortical neurons in response to somatic time-varying current injection [30] and includes two major features of cortical pyramidal cells: spike-frequency adaptation and realistic spike initiation [31]. As we expected, the model was still able to accurately estimate source location, with quantitatively similar results (Figure 9D–F).

### Learning

**Figure 10:**
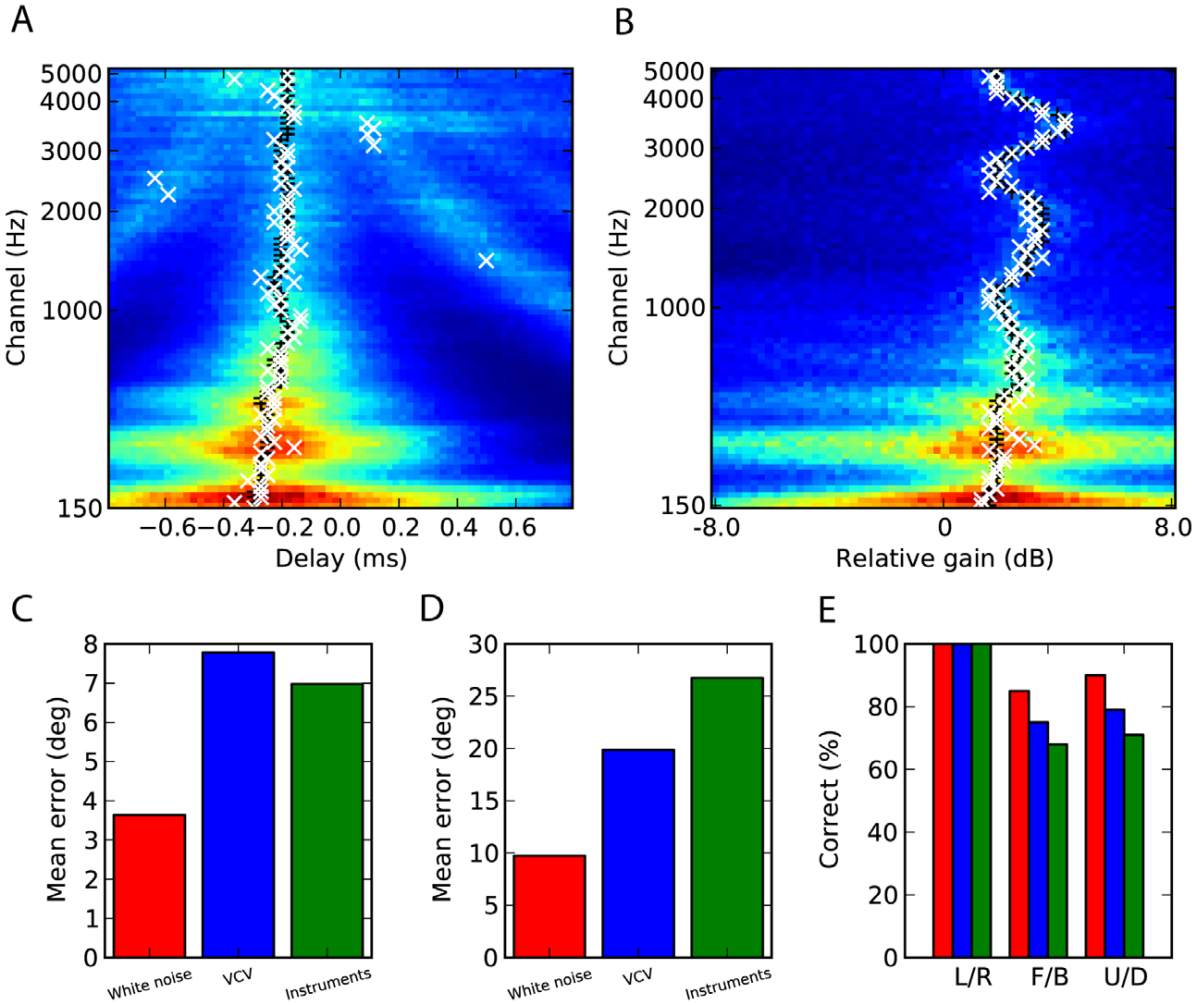
Learning delays and gains. (A, B) Response of a population of postsynaptic neurons with various preferred frequencies, interaural delays and gains to a long broadband sound (10 s) played at a particular location. (A) Maximum neural response (color-coded) over all the gains, for each frequency and relative delay. (B) Maximum neural response over all the delays, for each frequency and relative gain. In both (A) and (B), the white x symbols show the maximum response for each frequency, and the black+symbol shows the choice of best delay (A) and relative gain (B) for that location in the approximate model, based on the cross-correlation of HRIRs. (C, D, E) Performance of the approximate model (as in Figure 8) using delays and gains learned from hearing 7 example sounds of 1 s duration from each location.

In the model, source location is indicated by the activation of a specific neural assembly. Thus, estimating the source location requires that each physical location has been assigned to a neural assembly. We suggest that this assignment could be obtained through Hebbian learning, for example by associating neural activation with visual cues. In Figure 10, we show the response of a population of postsynaptic neurons with various preferred frequencies, interaural delays and gains to a long broadband sound (20 s) played at a particular location. Picking the maximally active neuron in each frequency channel (white crosses) defines a neural assembly that is indeed very close to the choice we previously made from the knowledge of HRTFs (black crosses). We estimated the performance of the model when the location-specific assemblies were learned in this way from 7 seconds of white noise played at each location (Figure 10, C–E). The error in azimuth, elevation and categorization was only slightly worse (compare with Figure 8, G–I). Note that the training data consisted of white noise while the test data included different types of sounds (speech and musical instruments).

**Figure 11:**
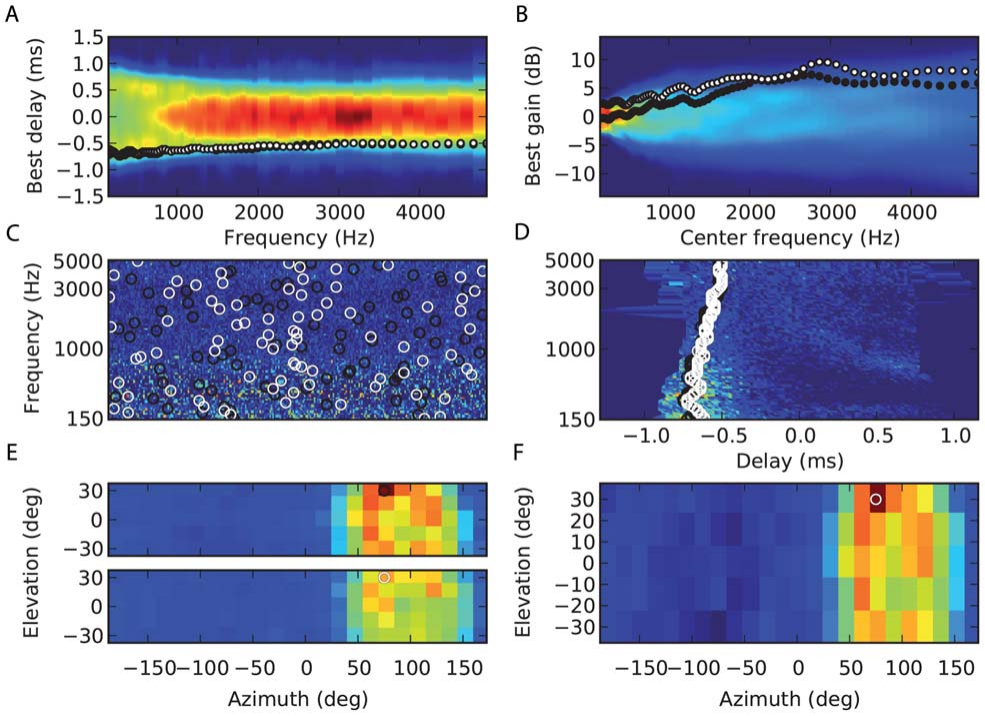
Simultaneous representation of two acoustical environments (two sets of HRTFs). (A, B) Best delay and relative interaural gain for two assemblies (black and white circles) corresponding to the same location in the two different HRTF sets, as in Figure 8E and F. (C, D) Response of the postsynaptic neurons to a sound at a particular location with one HRTF set, as in Figure 3C and D. The black circles show the neurons tuned to the correct location for one set, the white for the other. (E) Summed responses of location-specific assemblies for the two HRTF sets (each assembly has a preferred location and set), for a particular location and HRTF set. The circles represent the source location in the correct (black) and incorrect (white) set. The maximally activated assembly encodes for both location and HRTF set. (F) Summed responses of neural assemblies as a function of preferred location, for both sets (sum of the two sets of responses in E).

Since source location is encoded in the identity of (possibly overlapping) neural assemblies, learning consists in assigning a correct label to an assembly rather than in tuning parameters. Therefore, the same network can encode several acoustic environments: it is only the mapping from assembly activation to physical location that is environment-specific. Humans can learn this mapping when their acoustical cues change, for example when molds are inserted into their ears [32]–[34] (although these experiments mainly modify spectral cues rather than binaural cues). Interestingly, although learning a new mapping can take a long time (several weeks in the first study), the previous mapping is instantly recovered when the ear molds are removed, meaning that the representations of the two acoustical environments do not interfere, consistently with our model. We tested this idea by using two different sets of HRTFs (corresponding to two different human subjects) with the same network model. Figure 11A–D shows that the same source location activates two different neural assemblies depending on the HRTF set. We defined two mappings from neural activation to physical location, one for each HRTF set, by associating a neural assembly with each location, as previously. When a sound was presented through a particular HRTF set, it maximally activated a neural assembly assigned to the correct HRTF set at the correct location (Figure 11E), so that the model was still able to accurately estimate the source location (Figure 11F), as well as to identify the acoustical environment.

## Discussion

The acoustical transformation of a sound between source and ear is a linear filter that depends on their relative positions. The binaural stimulus resulting from the two ears receiving different filtered signals of the same source has a structure that is indicative of source location. We looked for correlates of this binaural structure in neuron models described by spectro-temporal filtering and spiking nonlinearity. We found that the *synchrony receptive field* of a pair of monaural neurons, defined as the set of stimuli that induce synchronous spiking in these neurons, defined a set of source locations (more precisely, filter pairs) independently of source signal. This is a very interesting property for source localization because an animal must be able to estimate the location of an unexpected sound. These location-specific synchrony patterns are then mapped to the activation of location-specific assemblies of postsynaptic neurons. The spiking model we implemented was indeed able to accurately locate a variety of sound sources in a virtual acoustic environment, and was robust to significant changes in model properties, including changes in the neuron models themselves, and to both physiological and acoustical noise. It demonstrates the computational relevance of relative spike timing to extract spatial information about sources independently of the source signal. In the model, source location is encoded into overlapping neural assemblies, and the mapping from neural activation to source location could be learned by Hebbian mechanisms (for example by association with visual cues). Because only this latter mapping depends on the acoustical environment, the model could simultaneously store several representations of different environments and both accurately estimate source location and identify acoustical environment.

### Cues for elevation

**Figure 12:**
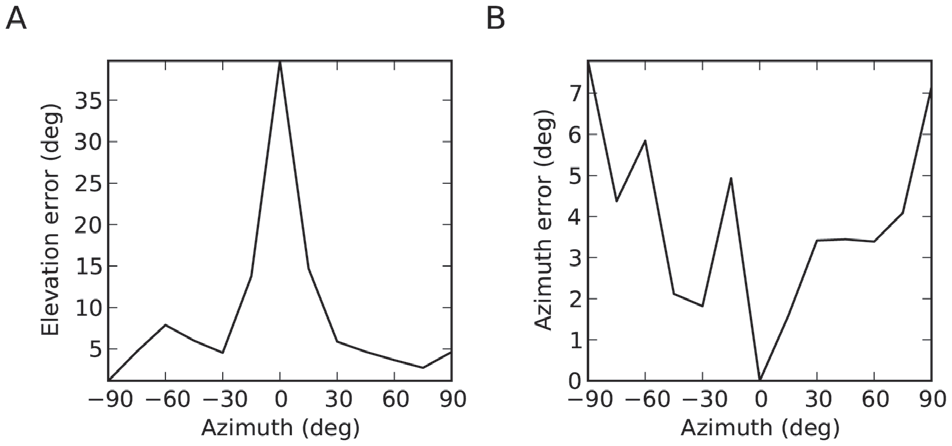
Mean error in estimates of elevation (A) and azimuth (B) of white noise in the approximate model as a function of the azimuth of the sound source. The elevation error at zero azimuth is close to the chance level of 36 degrees.

Our model only uses binaural cues, while it is known that the dominant cue for elevation is monaural spectral information in high frequencies [1], [35]. It may be surprising that the model can estimate elevation with only binaural cues, but psychophysical results show that human subjects can extract information about elevation from signals that are low-pass filtered below 3 kHz, where monaural spectral information is minimal [22], except in the median plane. Indeed, for any elevation in the median plane, the left and right signals are close to identical, therefore binaural cues do not provide information about elevation. Interestingly, we find the same pattern in our model (Figure 12A): performance in estimating elevation is close to chance level in the median plane. The azimuth error was also larger away from the median plane (Figure 12B), which is consistent with psychophysical experiments (see Figure 4A in [36]). Since the model does not use high-frequency monaural spectral information, it might be more relevant to compare psychophysical results with our model performance when both are constrained to the low-frequency range. Supplementary Figure S3 shows the performance of the approximate model when frequency ranges from 150 Hz to 3 kHz (as in reference [22]), which is close to the results shown earlier (Figure 8G–I). The same estimation error patterns were also seen in this case. It could be argued that the model indirectly uses monaural cues in the form of elevation-dependent spectral notches, but it only does so by comparing the two binaural signals rather than extracting spectral information from each monaural signal, which explains why it cannot estimate elevation in the median plane.

### Previous models

**Figure 13:**
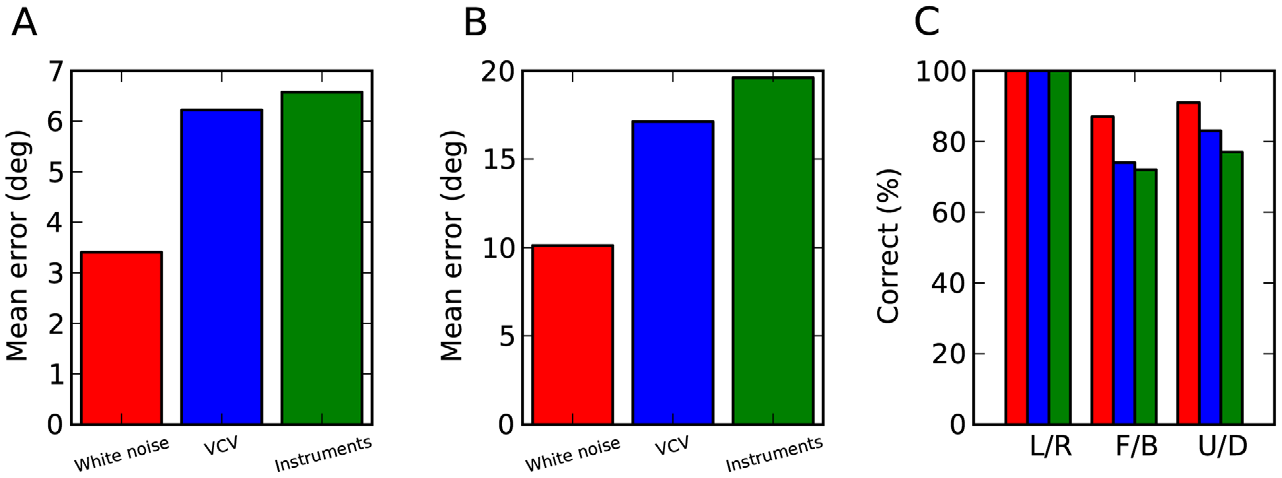
Performance of the model in low frequencies (under 3kHz). (A, B, C) Performance of the approximate model, as in Fig. 5G–I, for low frequencies only (80 channels distributed on the ERB scale from 150Hz to 3kHz).

Previous neural models focused on the mechanisms of cue extraction such as ITD sensitivity, using delayed tones or noise bursts (as in e.g. [13]), whereas we addressed the problem of estimating the location of arbitrary unknown sounds in a realistic acoustical environment. Since our model relies on coincidence detection between monaural inputs, it could be compared to the Jeffress model [6], where a neuron is maximally activated when acoustical and axonal delays match - although the Jeffress model is restricted to azimuth estimation. However, because in our model the inputs to these binaural neurons are precise spike trains rather than Poisson processes, the operation that they perform is more accurately described as a similarity operation (firing when the inputs are similar) than as a cross-correlation. In particular, this similarity operation includes level differences as well as timing differences. Indeed, there is considerable difficulty in implementing the Jeffress model with neuron models when realistic acoustical cues are considered, because ILDs always co-occur with ITDs and disturb spike timing. Figure 13A–C shows that model performance indeed drops when neural filtering is restricted to band-pass filtering and delays (no gains). In our model, the sensitivity of binaural neurons to ILDs comes from the fact that monaural neurons fire earlier as sound level increases, which is unavoidable if spikes are triggered when a threshold is reached. This “time-intensity trading” has been observed in the auditory nerve [37]: the effect is small but within the sensitivity of the binaural system. It has not yet been measured in bushy cells, which would correspond to the monaural neurons in our model (see below).

Several computational algorithms address the full problem of sound localization in a virtual acoustic environment with realistic sounds, but without a neural implementation. In our model, two monaural neurons fire in synchrony when the combinations of acoustical and neural filtering match on both sides. This is conceptually similar to the Equalisation-Cancellation (EC) model [18]–[20], in which compensating interaural gain and delay are chosen so that the two signals maximally match (i.e., by minimizing the difference). In the original EC model, this was done for the broadband signals but later versions of the model used multiple frequency bands [15]. Besides the fact that our model has a straightforward interpretation in terms of neural responses, we highlight two conceptual differences. Firstly, the signal transformations are not restricted in principle to delays and gains (as in our approximate model), but could include any sort of filtering (as in the ideal model). Although the performance of the approximate model was good, the ideal model was more accurate, in fact almost perfect when locations where restricted to the horizontal plane. When considering more complex environments (with reflections), the difference could be even more important. Secondly, our model provides an online, instantaneous estimation of source location, which could potentially change if the source moves. Additionally, the fact that the spike trains of the location-specific assemblies are locked to the signal may be useful to bind the spatial information with other types of information, for example to listen to a source at a particular location, or to integrate multimodal information (e.g. binaural information with visual motion). Recently, MacDonald [16] proposed a signal processing method (with no neural implementation) to localize sound sources in the horizontal plane that is conceptually very similar to our *ideal* model, where coincidence detection is replaced by Pearson correlation between the two transformed monaural signals. Interestingly, the estimation was found to be quite robust to background noise. Our model provides a neural implementation and works in multiple frequency channels rather than on the entire signal. It also provides signal-locked estimations. Perhaps more importantly, our *approximate* model provides a framework for learning to localize without explicit knowledge of HRTFs.

### Biological plausibility

The model we implemented includes the following components: acoustical environment, auditory periphery, and central neurons.

First, we modeled acoustical stimuli by using a variety of recorded sounds (noise, speech, musical instruments), and the acoustical environment was reproduced using human HRTFs, measured in anechoic conditions. This includes the diffraction of sounds by the head, pinnae and torso, which is much more realistic than using fixed ITDs and ILDs: even without considering the high frequency spectral notches introduced by the pinnae, ITDs are frequency-dependent for a given source location [21]. We also checked that model performance was robust to additional acoustical noise (Figure 9C). Thus, it can be considered as a reasonably realistic reproduction of the acoustical environment of humans in anechoic conditions. A more realistic model would include reflections, at least from the ground. If the delay between the direct sound and the reflection is large, then psychophysical studies suggest that the reflected sound is suppressed by the auditory system [38], but it would presumably require specific mechanisms, which we did not address. If the delay is short, it could change the binaural cues but because the physical laws of sound propagation are linear, their effect could still be modeled as location-dependent linear filtering, simply with different HRTFs than in the anechoic case. Therefore it should not impair the performance of the model, as long as the acoustical environment is known.

Our model of the auditory periphery is rather simple, compared to recent detailed models of auditory nerve fibers [39]. The main reason is practical. In the model we simulated up to 240 channels, with various sounds, each one played at all measured locations (azimuth and elevation, almost 200 locations), totaling up to 90,000 filters and 10^6^ neurons. Simulations took several days even after being accelerated using graphics processing units (GPUs). Using more realistic models is possible in principle, but would require faster hardware or substantially improved numerical techniques. The second reason is to keep the model simple enough to clearly demonstrate the underlying principle. Nonetheless, it includes the following ingredients: outer ear filtering (implicitly included in the HRTFs), band-pass filtering, compression, half-wave rectification, physiological and acoustical noise and spiking. Middle ear filtering was not included, but since it affects both ears equally (and therefore does not affect interaural differences), it should not have any impact on the performance of our model, which we checked with white noise stimuli (not shown). To generate spikes from the filtered signals, we used noisy neuron models (integrate-and-fire or more complex models in Figure 9D–F) rather than Poisson processes. These models implicitly include low pass filtering of the input signal (via the leak current). In Figure 13, we checked that the model also worked if sounds only contained frequencies below 3 kHz, where the temporal fine structure of sounds is still represented in the firing of auditory nerve fibers (we also found similar results for sounds below 1.5 kHz). To decompose sounds into frequency bands, we used gammatone filters. Other filters could be used, such as gammachirps, but the principle of the model does not rely on these details. Finally, the model included strong nonlinearities (half-wave rectification and compression) but they did not seem to affect the performance of the model.

There were two types of neurons in our model: monaural neurons and binaural neurons. In our description of the synchrony receptive field, we considered that the responses of monaural neurons consist of linear filtering followed by spiking nonlinearity. While this is clearly an approximation, it seems reasonable for the earliest neural structures in the auditory system. In the specific model we implemented, the strong nonlinearities in signal filtering (half-wave rectification and compression) did not seem to affect the principle of the model. We found that model estimations were still accurate when postsynaptic potentials were as long as about 4 ms, which is consistent with electrophysiological measurements of neurons in many structures in the auditory system [24]–[26]. The model was also robust to rather large levels of intrinsic noise (Figure 9A). However two assumptions restrict the set of candidate neural structures where these neurons could reside: neurons should be mainly monaural and their firing should be precisely time-locked to the stimulus. Most likely candidates are neurons in the cochlear nucleus, such as bushy cells. These cells are indeed essentially monaural and their spikes are precisely time-locked to sound stimuli [40], [41]. In the *approximate model*, we assumed that the receptive field of these neurons can be modeled as a band-pass filter (gammatone) with various gains and delays. Differences in input gains could simply arise from differences in membrane resistance, or in the number and strength of the synapses made by auditory nerve fibers. Delays could arise from many causes: axonal delays (either presynaptic or postsynaptic), cochlear delays [42], inhibitory delays [43]. In the *ideal model*, we assumed a larger diversity of receptive fields, in fact we assumed that all combinations of HRTFs and band-pass (gammatone) filters were represented, which might seem unrealistic. However, it does not mean that HRTFs themselves are represented in the auditory system. As is seen in Figure 5, these combined filters look very much like gammatone filters, but with variable envelopes. The variability of receptive fields of bushy cells could perhaps be characterized using reverse correlation techniques.

**Figure 14:**
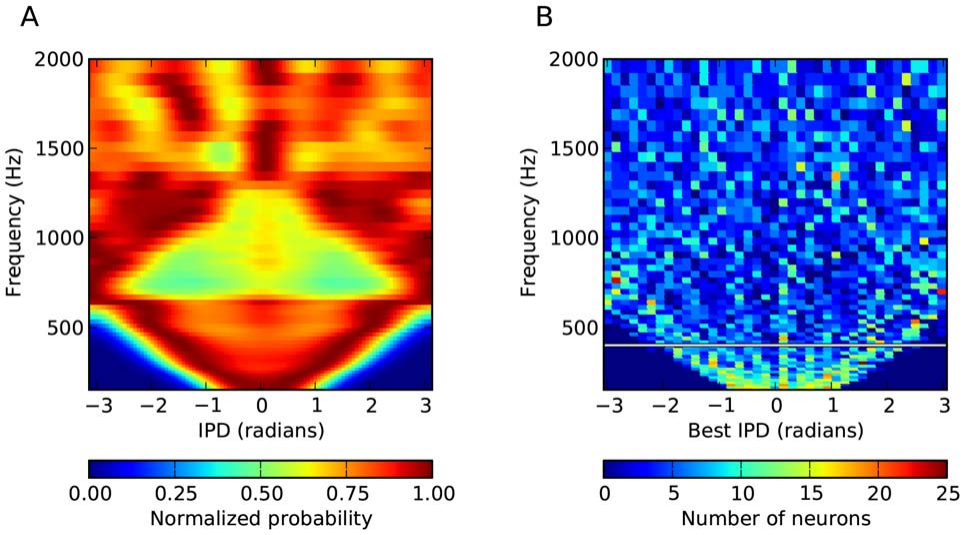
Distribution of interaural phase differences (IPD) in the binaural signals and preferred IPDs of binaural neurons. The color codes follow the conventions of Fig. 3 of Harper and McAlpine (2004). (A) Distribution of IPDs across all HRTFs (including all measured azimuths and elevations), as a function of frequency. The dark triangle corresponds to the physical limit of ITDs, which is smaller than half a period for low frequencies, while the light triangle corresponds to the situation when the maximum ITD is between π and 2π, which makes larger IPDs more represented than smaller ones (imagine folding a larger triangle at the vertical lines IPD = −π and IPD = π). (B) Distribution of best IPDs of neurons in the approximate model as a function of their preferred frequency. Best IPDs were measured using delayed white noise. The horizontal line at 400 Hz represents the frequency above which best delays should be continuously distributed, according to Harper and McAlpine (2004). A very similar distribution was obtained with the ideal model (not shown).

Spherical bushy cells project to binaural neurons in the medial superior olivary nucleus (MSO) [44]. Thus it seems natural to identify the binaural neurons in our model with these cells (the inferior colliculus (IC) and the dorsal nucleus of the lateral lemniscus (DNLL) also contain neurons with similar properties). In small mammals (guinea pigs, gerbils), it has been shown that the best phases of binaural neurons in the MSO and IC are scattered around ±π/4, in constrast with birds (e.g. barn owl) where the best phases are continuously distributed [45], [46]. In larger mammals such as cats, best IPDs in the MSO are more continuously distributed [47], with a larger proportion close to 0 (Figure 18 in [47]). It has not been measured in humans, but the same optimal coding theory that predicts the discrete distribution of phases in small mammals predicts that best delays should be continuously distributed above 400 Hz (80% of the frequency channels in our model). Figure 14 shows the distribution of best phases of binaural neurons in the approximate model as a function of preferred frequency (nearly identical results were obtained for the ideal model). It appears that the distribution is consistent with these predictions in humans (Figure 3 in [12]). However, one fact that is in contradiction with our model is that the best delays in both birds and mammals (including humans, based on fMRI studies [48]) are almost always smaller than half the characteristic period, i.e., they are within the π-limit. To check whether this was a critical element of our model, we estimated the performance of the approximate model when all best delays were constrained to the π-limit, and we found that it was essentially unchanged (Supplementary Figure S4). This is not very surprising since best delays above the π-limit are mostly redundant.

### Challenging sound localization problems

The HRTFs used in our virtual acoustic environment were recorded at a constant distance, so that we could only test the model performance in estimating the azimuth and elevation of a sound source. However, in principle, it should also be able to estimate the distance when the source is close (when the source is far, binaural cues are not informative of distance because the sound wave becomes a plane wave). A more difficult problem is that of reflections. In principle, our framework applies equally well to any acoustical environment, whether anechoic or not, but the mapping between neural assemblies and physical location must be known, meaning that the acoustical environment must be familiar. To estimate the location of a source in an unknown environment, one possibility would be to isolate the direct sound from the reflections, but this requires additional mechanisms, which probably underlie the precedence effect [38]. Finally, a challenging question is how the auditory system might perform this task in the presence of multiple sources or ambient noise [49], or use localization cues to listen to a particular source [50], [51]. In our model, binaural neurons respond only when their inputs receive consistent signals, so that spectro-temporal regions where noise dominates the signal should be ignored. Thus, we suggest that our model could address this more challenging task by “listening in the dips” of the noise to extract reliable information, in the same way as humans are thought to understand speech in noisy environments [52].

## Methods

All programming was done in the Python programming language, using the “Brian” spiking neural network simulator package [53]. Simulations were performed on Intel i7 Core processors with dual NVIDIA GTX295 graphics processing units (GPUs). Linear filtering was carried out in parallel on the GPUs with a custom algorithm designed for large filterbanks (around 30,000 filters in our simulations, or 90,000 in the simulations for Figure 8, J–L), reducing computation times for each sound from hours to minutes. The largest model (Figure 10) involved approximately one million simulated neurons. The overall structure and architecture of the model is illustrated in Figure 3.

### Virtual acoustics

Sound sources used were: broadband white noise; recordings of instruments and voices from the RWC Music Database (http://staff.aist.go.jp/m.goto/RWC-MDB/); recordings of vowel-consonant-vowel sounds [54]; and pure tones between 150 Hz and 5 kHz, uniformly distributed in ERB scale (Figure 4). All sounds were of 500 ms duration, cut to this length in the case of the VCVs (from around 600ms), and repeated twice in the case of the instruments (of length 250ms), and were presented at 80 dB SPL. Sounds were filtered by head-related impulse responses (HRIRs) from the IRCAM LISTEN HRTF Database (http://recherche.ircam.fr/equipes/salles/listen/index.html). HRIRs from this and other databases do not provide sufficiently accurate timing information at frequencies below around 150Hz, and so subsequent cochlear filtering was restricted to frequencies above this point.

### Cochlear and neural filtering

Head-filtered sounds were passed through a bank of fourth-order gammatone filters with center frequencies distributed on the ERB scale, modeling cochlear filtering [55], [56]. Additional linear filters were then applied: either the entire set of head filters (ideal model) or only gains and delays (approximate model). 80 channels were used in all models except for Figure 8, J–L, in which 240 channels were used. Center frequencies were chosen from 150Hz to 5kHz in all models except for Supplementary Figure S3, in which an upper limit of 3kHz was used.

#### Neuron model

The filtered sounds were half-wave rectified and compressed by a 1/3 power law 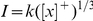 (where x is the sound pressure in pascals). The resulting signal was used as an input current to a leaky integrate-and-fire neuron with noise. The membrane potential V evolves according to the equation:
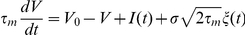
where τ_m_ is the membrane time constant, V_0_ is the resting potential, ξ(t) is Gaussian noise (such that 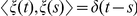) and σ is the standard deviation of the membrane potential in the absence of spikes. When V crosses the threshold V_t_ a spike is emitted and V is reset to V_r_ and held there for an absolute refractory period t_refrac_. These neurons make synaptic connections with binaural neurons in a second layer (two presynaptic neurons for each binaural neuron). These coincidence detector neurons are leaky integrate-and-fire neurons with the same equations but their inputs are synaptic. Spikes arriving at these neurons cause an instantaneous increase W in V (where W is the synaptic weight). Parameter values are given in Table 1.

**Table 1:** Neuron model parameters.

Parameter	Value	Description
V_r_	−60 mV	Reset potential
V_0_	−60 mV	Resting potential
V_t_	−50 mV	Threshold potential
t_refrac_	5 ms0 ms	Absolute refractory period(for binaural neurons)
σ	1 mV	Standard deviation of membrane potential due to noise
τ_m_	1 ms	Membrane time constant
W	5 mV	Synaptic weight for coincidence detectors
k	0.2 V/Pa^1/3^	Acoustic scaling constant

### Adaptive exponential integrate-and-fire (AdEx) neuron model

For Figure 8C–F, an *AdEx* neuron was used. We used the equations and parameters for a regular spiking *AdEx* neuron from [29], with an additional white noise current (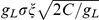). The leak conductance was adjusted so that the membrane time constant was 1 ms, as previously (giving g_L_ = 281 nS). The acoustical input to the encoder neurons was also scaled to provide the same input level as before, relative to threshold. The standard deviation of the noise and the synaptic weights were doubled so as to represent the same proportion of the distance between threshold and rest as in the previous model (σ = 2 mV and W = 10 mV). Finally, the model had the same refractory properties as in the previous model.

### Selecting gains and delays in the approximate model

For a given location and frequency channel with corresponding HRIRs L and R (after gammatone filtering), the gains (g_L_, g_R_) and delays (d_L_, d_R_) of the two presynaptic monaural neurons were chosen to minimize the RMS difference
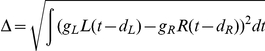
subject to the conditions max(g_L_, g_R_) = 1, d_L_≥0 and d_R_≥0. The RMS difference is minimized when the delays correspond to the maximum of the cross-correlation between L and R, 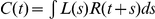, so that C(d_R_−d_L_) is the maximum, and 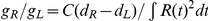.

### Estimating location from neural activation

Each location is assigned an assembly of coincidence detector neurons, one in each frequency channel. Each of these neurons has a pair of presynaptic neurons for which the synchrony field contains the given location (see Results). In the approximate model, this is obtained by selecting appropriate gains and delays for the presynaptic neurons as explained above; in the ideal model, the filters of the presynaptic neurons are the gammatone-filtered HRTFs for the given location. When a sound is presented to the model, the total firing rate of all neurons in each assembly is computed. The estimated location is the one assigned to the maximally activated assembly.

### Learning

In the model of learning shown in Figure 10, location-specific assemblies are learned by presenting unknown sounds at different locations to the model, where there is one coincidence detector neuron for each choice of frequency, relative delay and relative gain. Relative delays were uniformly chosen between −0.8ms and 0.8ms, and relative gains between −8 dB and 8 dB uniformly on a dB scale. In total 69 relative delays were chosen and 61 relative gains. With the 80 frequency channels, this gives a total of roughly 10^6^ neurons in the model. When a sound is presented at a given location, we define the assembly for this location by picking the maximally activated neuron in each frequency channel, as would be expected from a Hebbian learning process.

## Supporting Information

Figure S1 Confusion matrices for azimuth and elevation estimates. Confusion matrices for azimuth (A, C) and elevation (B, D) estimates for the ideal model (A, B) and the approximate model (C, D). The color of each square represents the probability that the model selects the location on the vertical axis given the source location on the horizontal axis.
(0.14 MB PDF)

Figure S2 Performance of the approximate and ideal models in the case when locations are constrained to the horizontal plane. Performance of the approximate and ideal models in the case when locations are constrained to the horizontal plane. (A) Mean error in azimuth estimation for the ideal model, as in Fig. 6G. (B) Categorization performance for the ideal model, as in Fig. 6I. (C, D) Same as A and B for the approximate model.
(0.16 MB PDF)

Figure S3 Performance of approximate model when neural filtering is restricted to bandpass-filtering and delays (no gains) or gains (no delays). Performance of approximate model when neural filtering is restricted to bandpass-filtering and delays (no gains; top row) or gains (no delays; bottom row). Colors and lines follow the same conventions as in Figure 6. In both cases, performance is significantly worse. (A,D) Error in azimuth. (B,E) Error in elevation. (C,F) Categorization performance (as in Figure 6).
(0.17 MB PDF)

Figure S4 Performance of approximate model when delays are constrained in the π-limit. Performance of approximate model when delays are constrained in the π-limit: in each band with central frequency f, all delays are replaced by delays between −1/(2f) and 1/(2f) with the same phases. Colors and lines follow the same conventions as in Figure 6. (A) Error in azimuth. (B) Error in elevation. (C) Categorization performance (as in Figure 6).
(0.14 MB PDF)
