# Supplementary material for: Spike-Timing-Based Computation in Sound Localization
Source: PLoS Comput Biol. 2010 Nov 11;6(11):e1000993. doi: 10.1371/journal.pcbi.1000993 (PMC2978676; doi:10.1371/journal.pcbi.1000993)
Supplement: Figure S1 — Confusion matrices for azimuth and elevation estimates. Confusion matrices for azimuth (A, C) and elevation (B, D) estimates for the ideal model (A, B) and the approximate model (C, D). The color of each square represents the probability that the model selects the location on the vertical axis given the source location on the horizontal axis. (0.14 MB PDF) [file pcbi.1000993.s001.pdf]

Supporting Information - Figure S1

Spike-timing-based computation in sound localization

Dan F. M. Goodman[1,2] and Romain Brette[1,2,*]
1, Laboratoire Psychologie de la Perception, CNRS and Université Paris Descartes, Paris, France
2, Département d'Etudes Cognitives, Ecole Normale Supérieure, Paris, France
* Email : romain.brette@ens.fr

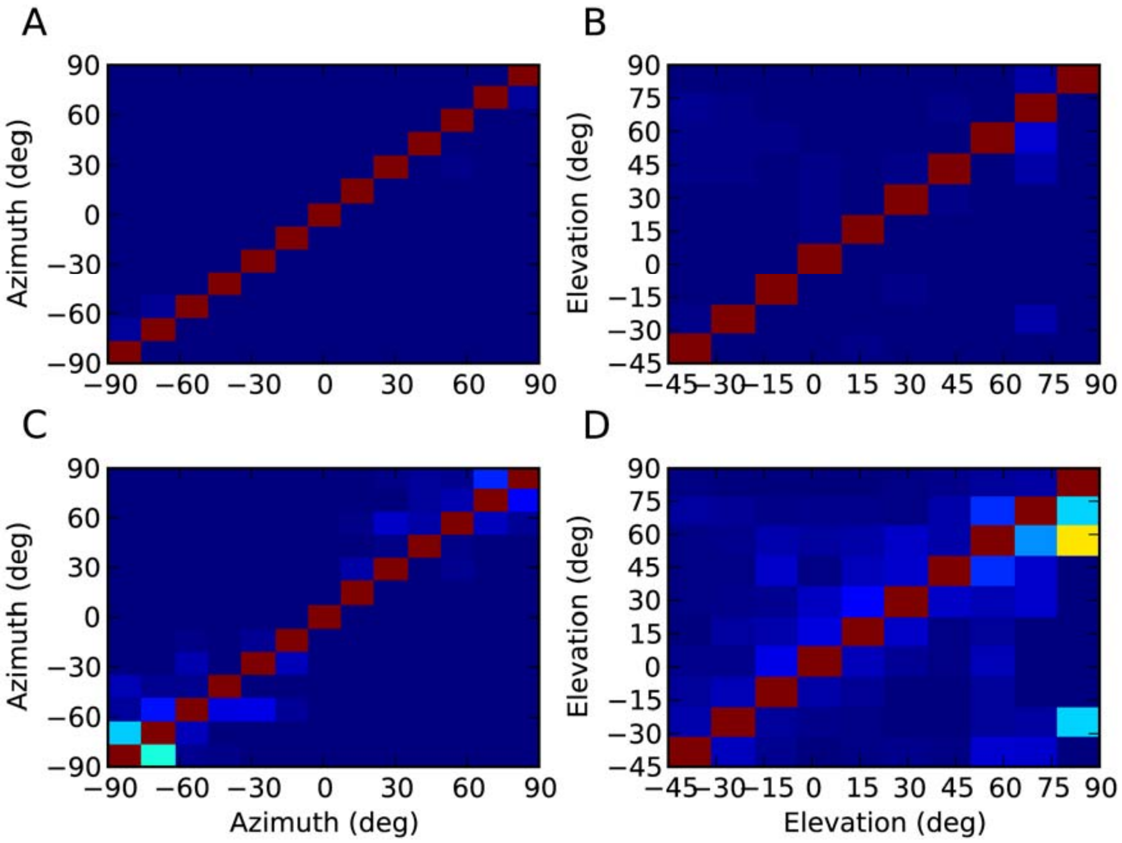

Figure S1. Confusion matrices for azimuth (A, C) and elevation (B, D) estimates for the ideal model (A, B) and the approximate model (C, D). The color of each square represents the probability that the model selects the location on the vertical axis given the source location on the horizontal axis.
