# Supplementary material for: Spike-Timing-Based Computation in Sound Localization
Source: PLoS Comput Biol. 2010 Nov 11;6(11):e1000993. doi: 10.1371/journal.pcbi.1000993 (PMC2978676; doi:10.1371/journal.pcbi.1000993)
Supplement: Figure S2 — Performance of the approximate and ideal models in the case when locations are constrained to the horizontal plane. Performance of the approximate and ideal models in the case when locations are constrained to the horizontal plane. (A) Mean error in azimuth estimation for the ideal model, as in Fig. 6G. (B) Categorization performance for the ideal model, as in Fig. 6I. (C, D) Same as A and B for the approximate model. (0.16 MB PDF) [file pcbi.1000993.s002.pdf]

Supporting Information - Figure S2

Spike-timing-based computation in sound localization

Dan F. M. Goodman[1,2] and Romain Brette[1,2,*]
1, Laboratoire Psychologie de la Perception, CNRS and Université Paris Descartes, Paris, France
2, Département d'Etudes Cognitives, Ecole Normale Supérieure, Paris, France
* Email : romain.brette@ens.fr

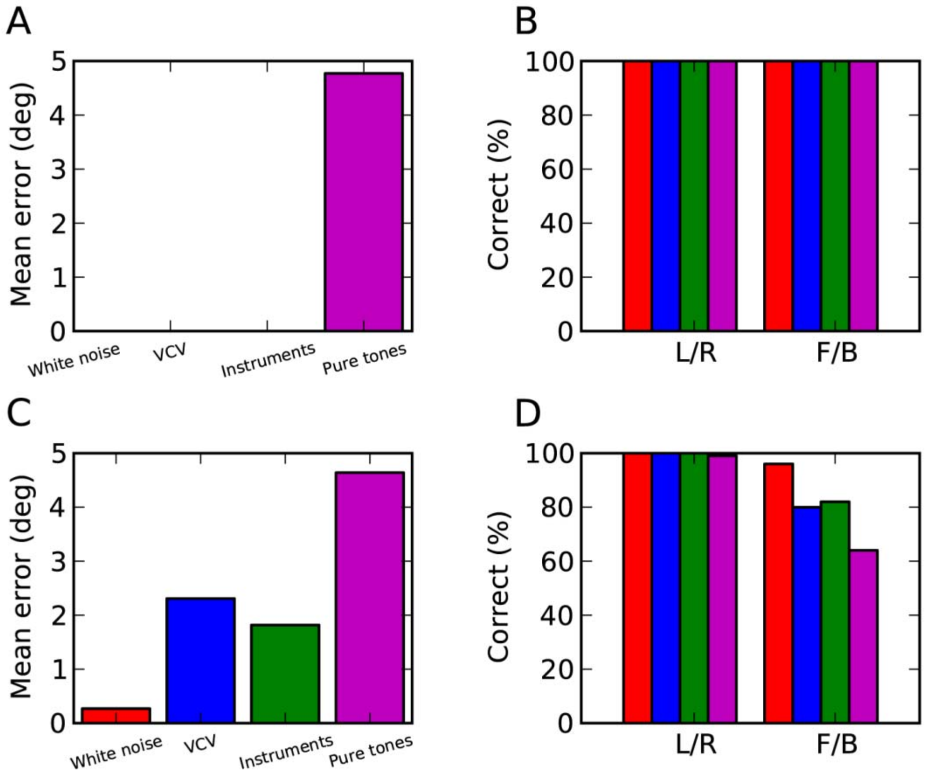

Figure S2. Performance of the approximate and ideal models in the case when locations are constrained to the horizontal plane. (A) Mean error in azimuth estimation for the ideal model, as in Fig. 6G. (B) Categorization performance for the ideal model, as in Fig. 6I. (C, D) Same as A and B for the approximate model.
