# Supplementary material for: Spike-Timing-Based Computation in Sound Localization
Source: PLoS Comput Biol. 2010 Nov 11;6(11):e1000993. doi: 10.1371/journal.pcbi.1000993 (PMC2978676; doi:10.1371/journal.pcbi.1000993)
Supplement: Figure S3 — Performance of approximate model when neural filtering is restricted to bandpass-filtering and delays (no gains) or gains (no delays). Performance of approximate model when neural filtering is restricted to bandpass-filtering and delays (no gains; top row) or gains (no delays; bottom row). Colors and lines follow the same conventions as in Figure 6. In both cases, performance is significantly worse. (A,D) Error in azimuth. (B,E) Error in elevation. (C,F) Categorization performance (as in Figure 6). (0.17 MB PDF) [file pcbi.1000993.s003.pdf]

Supporting Information - Figure S3

Spike-timing-based computation in sound localization

Dan F. M. Goodman[1,2] and Romain Brette[1,2,*]
1, Laboratoire Psychologie de la Perception, CNRS and Université Paris Descartes, Paris, France
2, Département d'Etudes Cognitives, Ecole Normale Supérieure, Paris, France
* Email : romain.brette@ens.fr

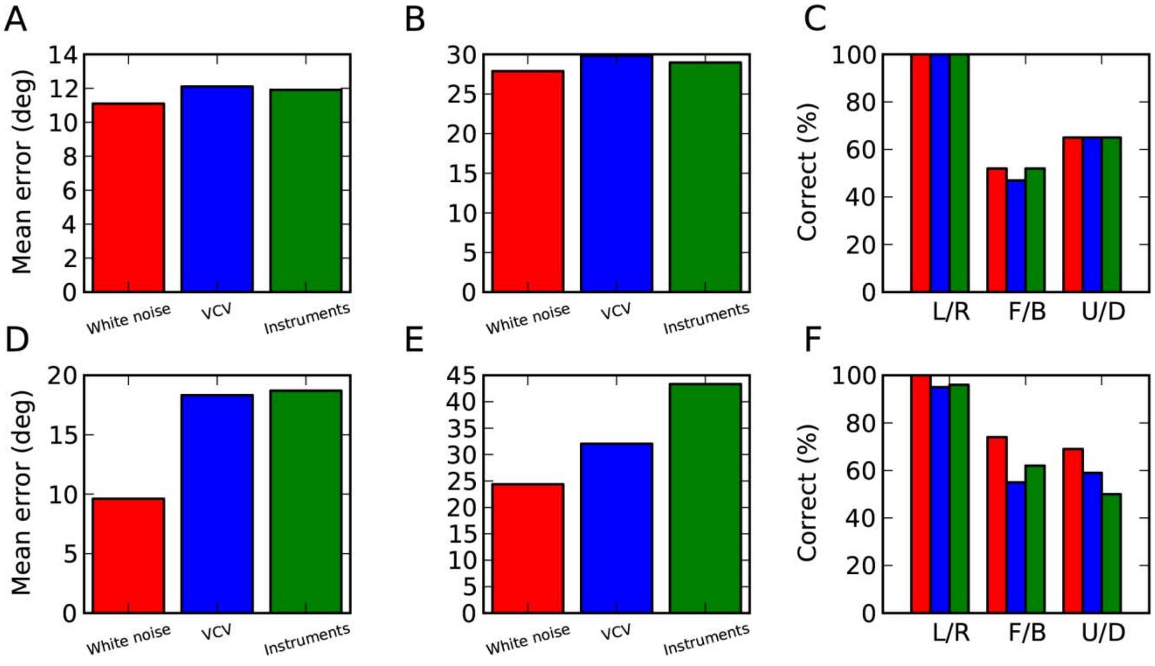

Figure S3. Performance of approximate model when neural filtering is restricted to bandpass-filtering and delays (no gains; top row) or gains (no delays; bottom row). Colors and lines follow the same conventions as in Figure 6. In both cases, performance is significantly worse. (A,D) Error in azimuth. (B,E) Error in elevation. (C,F) Categorization performance (as in Figure 6).
