# Supplementary material for: Spike-Timing-Based Computation in Sound Localization
Source: PLoS Comput Biol. 2010 Nov 11;6(11):e1000993. doi: 10.1371/journal.pcbi.1000993 (PMC2978676; doi:10.1371/journal.pcbi.1000993)
Supplement: Figure S4 — Performance of approximate model when delays are constrained in the π-limit. Performance of approximate model when delays are constrained in the π-limit: in each band with central frequency f, all delays are replaced by delays between −1/(2f) and 1/(2f) with the same phases. Colors and lines follow the same conventions as in Figure 6. (A) Error in azimuth. (B) Error in elevation. (C) Categorization performance (as in Figure 6). (0.14 MB PDF) [file pcbi.1000993.s004.pdf]

Supporting Information - Figure S4

Spike-timing-based computation in sound localization

Dan F. M. Goodman[1,2] and Romain Brette[1,2,*]
1, Laboratoire Psychologie de la Perception, CNRS and Université Paris Descartes, Paris, France
2, Département d'Etudes Cognitives, Ecole Normale Supérieure, Paris, France
* Email : romain.brette@ens.fr

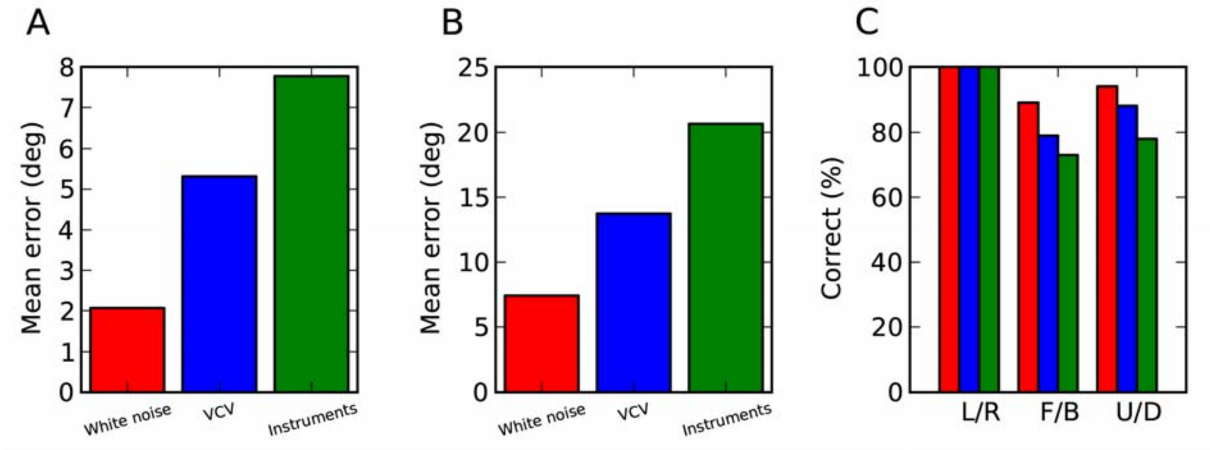

Figure S4. Performance of approximate model when delays are constrained in the π-limit: in each band with central frequency *f*, all delays are replaced by delays between *-1/(2f)* and *1/(2f)* with the same phases. Colors and lines follow the same conventions as in Figure 6. (A) Error in azimuth. (B) Error in elevation. (C) Categorization performance (as in Figure 6).
